# Improved Local Ternary Patterns for Automatic Target Recognition in Infrared Imagery

**DOI:** 10.3390/s150306399

**Published:** 2015-03-16

**Authors:** Xiaosheng Wu, Junding Sun, Guoliang Fan, Zhiheng Wang

**Affiliations:** 1 School of Computer Science and Technology, Henan Polytechnic University, 2001 Century Avenue, Jiaozuo 454000, China; E-Mails: wuxs@hpu.edu.cn (X.W.); wangzh@hpu.edu.cn (Z.W.); 2 School of Electrical and Computer Engineering, Oklahoma State University, 202 Engineering South, Stillwater, OK 74078, USA; E-Mail: guoliang.fan@okstate.edu

**Keywords:** local ternary pattern (LTP), robust LTP (RLTP), orthogonal combination of LTP (OC_LTP), automatic target recognition (ATR), soft concave-convex partition (SCCP)

## Abstract

This paper presents an improved local ternary pattern (LTP) for automatic target recognition (ATR) in infrared imagery. Firstly, a robust LTP (RLTP) scheme is proposed to overcome the limitation of the original LTP for achieving the invariance with respect to the illumination transformation. Then, a soft concave-convex partition (SCCP) is introduced to add some flexibility to the original concave-convex partition (CCP) scheme. Referring to the orthogonal combination of local binary patterns (OC_LBP), the orthogonal combination of LTP (OC_LTP) is adopted to reduce the dimensionality of the LTP histogram. Further, a novel operator, called the soft concave-convex orthogonal combination of robust LTP (SCC_OC_RLTP), is proposed by combing RLTP, SCCP and OC_LTP Finally, the new operator is used for ATR along with a blocking schedule to improve its discriminability and a feature selection technique to enhance its efficiency Experimental results on infrared imagery show that the proposed features can achieve competitive ATR results compared with the state-of-the-art methods.

## 1. Introduction

Automatic target recognition (ATR) is an important and challenging problem for a wide range of military and civilian applications. Since forward-looking infrared (FLIR) images are frequently used in ATR applications, many algorithms have been proposed in FLIR imagery in recent years [1], such as learning-based [2,3] and model-based [4–9] methods. Furthermore, there are also many hybrid vision-based approaches that combine learning-based and model-based ideas for object tracking and recognition in visible band images [10–12]. The advances in target detection and tracking in FLIR imagery and the performance evaluation work for the ATR system are referred to in [13] and [14], respectively.

Different from the learning-based, model-based and hybrid vision-based algorithms, Patel *et al.* introduced sparse representation-based classification (SRC) [15] into infrared ATR in [16], and the experimental results show that it outperforms the traditional ones with promising results.

As one of the learning approaches, the ATR task has also been cast as a texture analysis problem due to rich texture characteristics in most infrared imagery. Various texture-based ATR methods have been proposed in recent years [17,18]. In this paper, we focus on local binary pattern (LBP), a simple yet effective approach, for infrared ATR. It also has achieved promising results in several ATR applications in recent years, such as maritime target detection and recognition in [19], infrared building recognition in [20], ISAR-based ATR in [21] and infrared ATR in our previous work [22].

The LBP operator was firstly proposed by Ojala *et al.*, in [23], and it has been proven to be a robust and computationally simple approach to describe local structures. In recent years, the LBP operator has been extensively exploited in many applications, such as texture analysis and classification, face recognition, motion analysis, ATR and medical image analysis [24]. Since Ojala' s original work [23], the LBP methodology has been developed with a large number of extensions in different fields, such as the extensions from the viewpoint of improving the neighborhood topology [25–30], the extensions from the viewpoint of reducing the impact of noise [31–34], the extensions from the perspective of reducing the feature dimensionality [25,35,36], the extensions from the viewpoint of improving the encoding methods [22,37–42] and the extensions from the perspective of obtaining rotation invariant property [25,43–46].

More specifically, we are interested in the applicability of the local ternary pattern (LTP) [31] and the concave-convex partition (CCP) [22] for infrared ATR. The reason is that the LTP is robust to image noise, and it has been proven to be effective for infrared ATR. Additionally, the CCP can greatly improve the performance of the LTP in ATR [22]. In this work, we make several important improvements to further enhance the performance of LTP and CCP. First, we propose a robust LTP (RLTP) to reduce the sensitivity of LTP to the illumination transformation. Second, we develop soft CCP (SCCP) to overcome the rigidity of CCP. Third, the scheme of the orthogonal combination of local binary patterns (OC_LBP) [36] and a feature selection method [47] are introduced to reduce the dimensionality of the feature. Based on RLTP, SCCP and OC_LBP, a novel operator is introduced in the paper, which is named the soft concave-convex orthogonal combination of robust local ternary patterns (SCC_OC_RLTP). In addition, we also introduce a simple, yet effective blocking technique to further improve the feature discriminability for infrared ATR. Finally, we evaluate the newly-proposed operator with the sCCLTP (spatial concave-convex partition based local ternary pattern) [22] and the latest sparsity-based ATR algorithm proposed in [16]. Experimental results show that the presented method gives the best performance among the state-of-the-art methods.

The rest of the paper is organized as follows. We first briefly review the background of the basic LBP, LTP and OC_LBP. Then, we present the detailed feature extraction step, followed by the extensive experimental results on the texture databases and the ATR database. Finally, we provide some concluding remarks.

## 2. Brief Review of LBP-Based Methods

In this section, we only give a brief introduction of the basic LBP and its extensions, LTP and OC_LBP.

### 2.1. Local Binary Pattern

The basic LBP operator is first introduced in [23] for texture analysis. It works by thresholding a neighborhood with the gray level of the central pixel. The LBP code is produced by multiplying the thresholded values by weights given by powers of two and adding the results in a clockwise way. It was extended to achieve rotation invariance, optional neighborhoods and stronger discriminative capability in [25]. For a neighborhood (*P,R*), the basic LBP is commonly referred to as LBP*_P,R_*, and it is written as:

(1) $${\text{LBP}}_{P,R}=\sum_{i=0}^{P-1}s\left(p_{i}-p_{c}\right)\times 2^{i},s\left(x\right)=\{\begin{array}{cc} 1 & \text{if}\;x\geq 0\\ 0 & \text{otherwise} \end{array}$$

where *P* is the number of the sampling pixels on the circle, *R* is the radius of the circle, *p*_c_ corresponds to the gray value of the central pixel and *p_i_* corresponds to the gray value of each sampling pixel on the circle. In order to extract the most fundamental structure and rotation invariance patterns from LBP, the uniform and rotation invariant operator ${\text{LBP}}_{P,R}^{\text{riu}2}$ [25] is given as:

(2) $${\text{LBP}}_{P,R}^{\text{riu}2}=\{\begin{array}{ll} \sum_{i=0}^{P-1}s\left(p_{i}-p_{c}\right) & \text{if}\;U\left({\text{LBP}}_{P,R}\right)\leq 2\\ P+1 & \text{otherwise} \end{array}$$

where the superscript riu2 refers to the rotation invariant uniform patterns that have a *U* value (*U* ≤ 2). The uniformity measure *U* corresponds to the number of transitions from zero to one or one to zero between successive bits in the circular representation of the binary code LBP*_P,R_*, which is denned as:

(3) $$U\left({\text{LBP}}_{P,R}\right)=\left|s\left(p_{P-1}-p_{c}\right)-s\left(p_{0}-p_{c}\right)\right|+\sum_{i=1}^{P-1}\left|s\left(p_{i}-p_{c}\right)-s\left(p_{i-1}-p_{c}\right)\right|$$

All nonuniform patterns are classified as one pattern for ${\text{LBP}}_{P,R}^{\text{riu}2}$. The mapping from LBP*_P,R_* to ${\text{LBP}}_{P,R}^{\text{riu}2}$, which has *P* + 2 distinct output values, can be implemented with a lookup table.

### 2.2. Local Ternary Pattern

**Figure 1.**
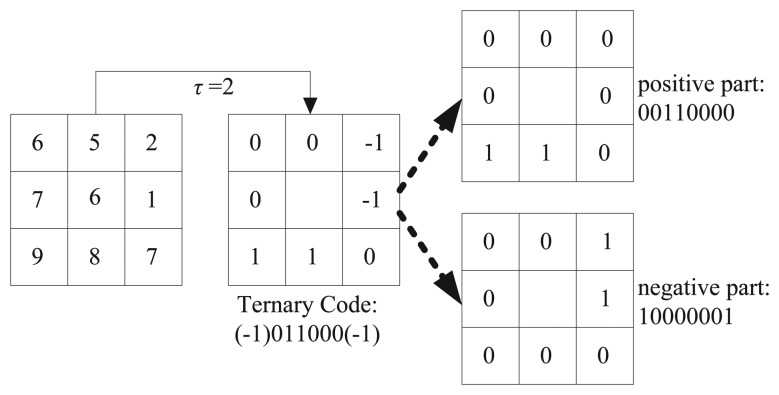
Calculation of the LTP with eight neighboring pixels.

The LBP is sensitive to noise, because a small gray change of the central pixel may cause different codes for a neighborhood in an image, especially for the smooth regions. In order to overcome such a flaw, Tan and Triggs [31] extended the basic LBP to a version with three-value codes, which is called the local ternary pattern (LTP). In LTP, the indicator *s* (*x*) is further defined as:

(4) $${\text{LTP}}_{P,R,\tau }=\sum_{i=0}^{P-1}s\left(p_{i}-p_{\text{c}}\right)\times 3^{i},s\left(x\right)=\{\begin{array}{ll} 1 & x\geq \tau \\ 0 & \left|x\right|<\tau \\ -1 & x\leq -\tau \end{array}$$

where *τ* is a threshold specified by the user. In order to reduce the feature dimension, a coding scheme is also represented by Tan and Triggs [31] by splitting each ternary pattern into two parts: the positive part and the negative part, as illustrated in Figure 1. Though the LTP codes are more resistant to noise, it is no longer strictly invariant to gray-level transformations, because *τ* is constant in feature extraction for all neighborhoods and all images in the database.

### 2.3. Orthogonal Combination of Local Binary Patterns

**Figure 2.**
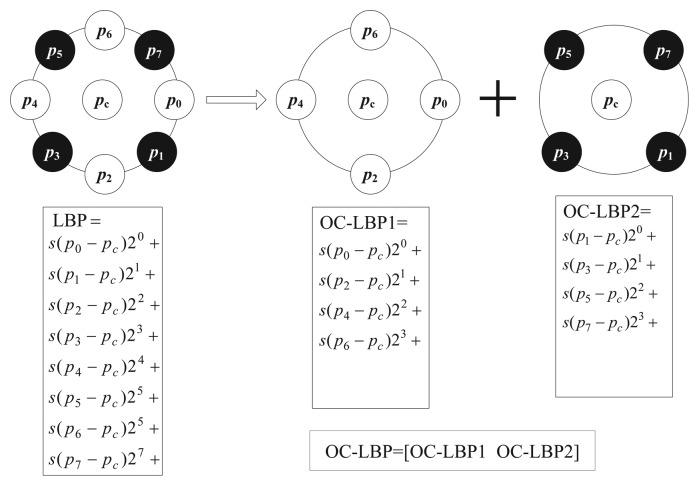
Calculation of local binary patterns (LBP) and the orthogonal combination of local binary patterns (OC_LBP) with eight neighboring pixels.

In [36], Zhu *et al.* proposed the orthogonal combination of local binary patterns (OC_LBP), which drastically reduces the dimensionality of the original LBP histogram to 4 × *P* by combining the histograms of [P/4]different four-orthogonal neighbor operators. Experimental results given in [36] show that OC_LBP is better than uniform patterns ${\text{LBP}}_{P,R}^{U2}$ in [25]. Figure 2 gives the comparison of calculating LBP and OC_LBP with eight neighboring pixels.

## 3. Feature Extraction

LTP and CCP have been proven to be robust for ATR in our previous work [22]. We also adopt them for feature descriptions in the paper. Furthermore, the robust local ternary patterns (RLTP) and soft concave-convex partition (SCCP) are presented to solve the flaws of LTP and CCP, respectively.

### 3.1. Robust Local Ternary Patterns

For LTP, it is not invariant to the gray-level transformations, because the threshold τ is a constant for all neighborhoods. Instead of employing a fixed threshold, we propose a robust method to assign its value based on the average gray value of the neighborhood. Let ω(*i*, *j*) be a neighborhood centered at pixel (*i*, *j*) in an image, *p*_*i*,*j*_ be the gray value of the pixel (*i*, *j*) and μ_*i*,*j*_ rage gray value of ω(*i*, *j*). Specifically, the new threshold τ_*i*,*j*_ or the neighborhood ω(*i*, *j*) is defined as follows:

(5) $$\tau_{i,j}=\left\lfloor \alpha \times \mu_{i,j}\right\rfloor$$

where α is a scaling factor and μ*_i,j_* is defined as:

(6) $$\mu_{i,j}=\frac{1}{P+1}\left(p_{i,j}+\sum_{k=0}^{P-1}p_{k}\right)$$

It is evident that the threshold τ*_i,j_* changes with the variation of the gray levels of the neighborhood ω(*i*, *j*). Therefore, it can help the LTP to retain the invariance with respect to illumination transformation. In this case, the robust LTP (RLTP) is given as:

(7) $${\text{RLPT}}_{P,R,\tau_{i,j}}=\sum_{k=0}^{P-1}\left(p_{k}-p_{i,j}\right)\times 3^{k},s\left(x\right)=\{\begin{array}{ll} 1 & x\geq \tau_{i,j}\\ 0 & \left|x\right|<\tau_{i,j}\\ -1 & x\leq -\tau_{i,j} \end{array}$$

### 3.2. Soft Concave-Convex Partition

It has been shown that the neighborhoods with different visual perceptions may have the same binary code by the LBP-based operators, and the concave-convex partition (CCP) was proposed to solve such a flaw in [22]. For simplicity, the average gray value (μ) of the whole image is chosen as a threshold to partition all of the neighborhoods into two categories, the concave and convex category. If μ*_i,j_* < μ, the neighborhood falls into the concave category, or else, it is classified as the convex category. It can be seen that the classification results depend entirely on the threshold μ. for CCP. Therefore, such a classification is a rigid partition. In this paper, we introduced a soft concave-convex partition (SCCP) definition as follows to overcome its shortcoming.

Given β as a scaling factor, if μ*_i,j_* < (1 − β) × μ, the central pixel (*i*, *j*) is regarded as a concave pixel and the neighborhood ω(*i*, *j*) as a concave neighborhood. If μ*_i,j_* ≥ (1 + β) × μ, the central pixel (*i*, *j*) is regarded as a convex pixel and ω(*i*, *j*) as a convex neighborhood. When β = 0, the SCCP reduces to the CCP.

### 3.3. Orthogonal Combination of Robust Local Ternary Patterns Based on SCCP

**Figure 3.**
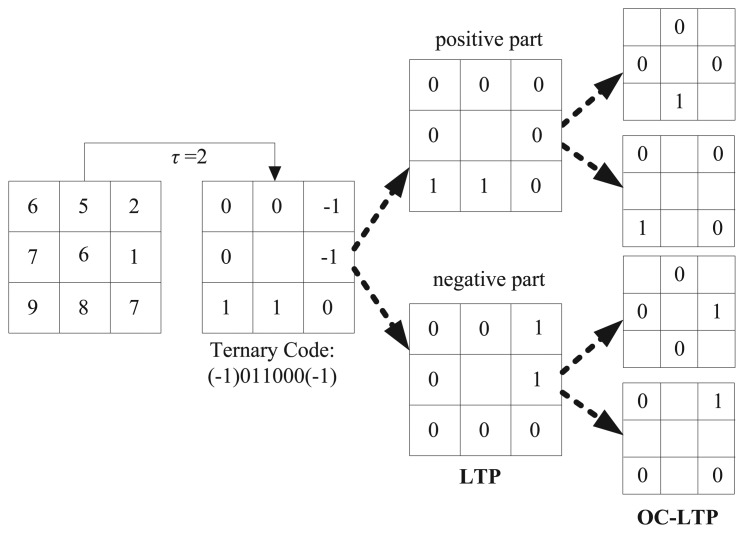
Calculation of the OC_LTP operators with eight neighboring pixels.

Based on OC_LBP and LTP, the orthogonal combination of local ternary patterns (OC_LTP) is proposed firstly in the paper. Figure 3 gives a calculation example for an eight-pixel neighborhood. Furthermore, OC_LTP is enhanced by the RLTP and SCCP. The new approach is named the soft concave-convex orthogonal combination of robust local ternary patterns (SCC_OC_RLTP). Table 1 gives the dimensionality comparison of OC_LBP, OC_RLTP and SCC_OC_LTP.

### 3.4. Blocking Methods

**Figure 4.**
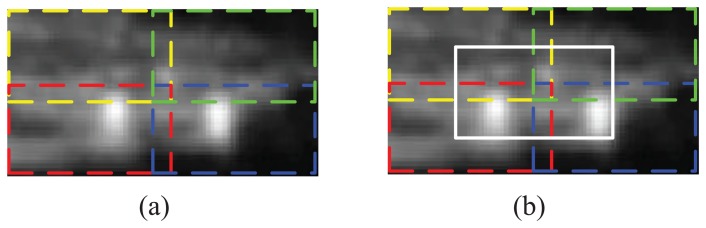
Two blocking methods to divide an infrared chip into multiple segments. (**a**) The chip is divided into four overlapped quadrants; (**b**) The chip is divided into five overlapped regions.

According to the report in [22] and [48], it is better to divide the infrared image into patches and to combine the feature of each patch together for higher performance. Six different blocking methods have been tested in our previous work [22], and the results show that the blocking method (illustrated in Figure 4a), which divides a chip into four quadrants that are slightly overlapped, gives more promising results. Because the objects are basically located in the center of the infrared image, we choose the center region as an additional block in this paper, which is illustrated in Figure 4b. After that, the features of the five blocks and that of the whole image are concentrated together for the image description.

### 3.5. Feature Selection

In our previous work [22], three different multi-resolutions, *P* = 8 and *R* = 1, *P* = 16 and *R* = 2 and *P* = 24 and *R* = 3, are combined together for feature description. Obviously, this leads to a sharp increase of the feature dimensionality. For sCCLTP [22], the dimensionality is 1080 bins ((104 + 72 + 40) × 5 = 1080); while, for the novel operator SCC_OC_RLTP, its dimensionality reaches to 4608 bins ((384 + 256 + 128) × 6 = 4608).

A tremendous amount of previous studies have demonstrated that a highly redundant feature set should have an intrinsic dimensionality much smaller than the actual dimensionality of the original feature space [49]. Namely, many features might have no essential contributions to characterize the datasets, and the features that do not affect the intrinsic dimensionality could be dropped. There are two general approaches of feature reduction, which include feature selection and feature recombination. The former method chooses a subset of original feature set just like the feature filter to achieve feature reduction, e.g., ${\text{LBP}}_{P,R}^{U2}$ in [25], the method based on differential evolution [47] (called FSDE in the paper) and discriminative features [35]. The latter obtains a new smaller feature set by a weighted recombination of the original feature set, e.g., independent component analysis (ICA), principal component analysis (PCA) and their improvements. In this paper, we performed the feature selection step to get a discriminative features subset from the original high dimensional features. To reach this goal, our interest focus on the FSDH in [47] for its promising results in feature selection.

### 3.6. Dissimilarity Measure

Various metrics have been presented to evaluate the dissimilarity between two histograms. As most LBP-based algorithms, we also chose the chi-square distance as the dissimilarity measure, which is defined as:

(8) $$d\left(H,B\right)=\sum_{i=1}^{K}\frac{{\left(h_{i}-b_{i}\right)}^{2}}{h_{i}+b_{i}}$$

where *H* = { *h_i_* } and *B* = {*b_i_*} (*i* = 1, 2,…, *K*) denote the two feature histograms and *K* is the number of bins in the histogram.

## 4. Experiments and Discussions

In this section, we first evaluate and compare LTP [31] and CCLTP [22], along with the improved methods, RLTP and SCCLTP (Soft Concave-Convex LTP), respectively, for texture classification. Then, we focus on OC_LBP, OC_LTP, CC_OC_LTP, OC_RLTP and SCC_OC_RLTP to examine their effectiveness for infrared ATR.

### 4.1. Experiments for Texture Classification

For texture classification, we chose the Outex database [50], which has been widely used for the comparison of LBP-based methods, as the test beds. For the Outex database, we chose Outex_TC_0010 (TC10) and Outex_TC_0012 (TC12), where TC10 and TC12 contain the same 24 classes of textures collected under three different illuminants (“horizon”, “inca” and “tl84”) and nine different rotation angles (0°, 5°, 10°, 15°, 30°, 45°, 60°, 75° and 90°). There are 20 non-overlapping 128 × 128 texture samples for each class under each situation. For TC10, samples of illuminant ‘inca’ and an angle of 0° in each class were used for classifier training, and the other eight rotation angles with the same illumination were used for testing. Hence, there are 480 (24 × 20) models and 3840 (24 × 8 × 20) validation samples. For TC12, all of the 24 × 20 × 9 samples captured under illumination “tl84” or “horizon” were used as the test data.

**Figure 5.**
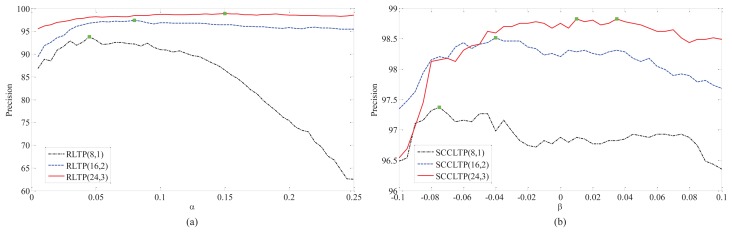
The curve of precision *vs.* α and β on TC10. (**a**) The results of RLTP(8,1), RLTP(16,2) and RLTP(24,3). (**b**) The results of SCCLTP(8,1), SCCLTP(16,2) and SCCLTP(24,3).

**Figure 6.**
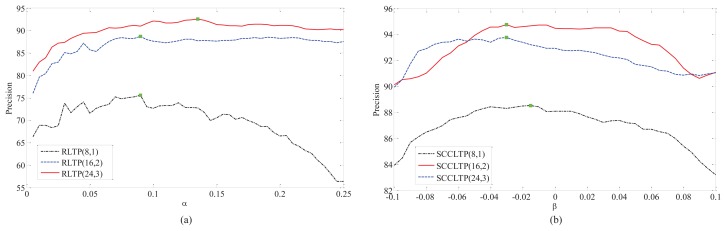
The curve of precision *vs.* α and β on TC12_001. (**a**) The results of RLTP(8,1), RLTP(16,2) and RLTP(24,3). (**b**) The results of SCCLTP(8,1), SCCLTP(16,2) and SCCLTP(24,3).

In this experiments, we firstly test the influence of α and β on RLTP and SCCLTP. For TC12, the samples captured under illumination “horizon” (TC12_001) were used as the test data. The curves of precision *vs.* α and β on TC10 and TC12_001 are shown in Figures 5 and 6, where RLTP(8,1), RLTP(16,2) and RLTP(24,3) denote ${\text{RLTP}}_{8,1}^{\text{riu}2}$, ${\text{RLTP}}_{16,2}^{\text{riu}2}$, and ${\text{RLTP}}_{24,3}^{\text{riu}2}$, and SCCLTP(8,1), SCCLTP(16,2) and SCCLTP(24,3) denote ${\text{SCCLTP}}_{8,1}^{\text{riu}2}$, ${\text{SCCLTP}}_{16,2}^{\text{riu}2}$ and ${\text{SCCLTP}}_{24,3}^{\text{riu}2}$, respectively. The colored boxes in the curves of different methods in Figures 5 and 6 denote such methods obtaining the best performance at those points. It can be seen that the optimal values of α and β are different for *P* = 8 and *R* = 1, *P* = 16 and *R* = 2 and *P* = 24 and *R* = 3. The results in Figure 5b show that the features ${\text{SCCLTP}}_{8,1}^{\text{riu}2}$ and ${\text{SCCLTP}}_{16,2}^{\text{riu}2}$ get the best performance when β < 0. While, the feature ${\text{SCCLTP}}_{24,3}^{\text{riu}2}$ gets the best performance when β > 0. The results in Figure 6b show that the three features, ${\text{SCCLTP}}_{8,1}^{\text{riu}2}$, ${\text{SCCLTP}}_{16,2}^{\text{riu}2}$ and ${\text{SCCLTP}}_{24,3}^{\text{riu}2}$, achieve the best performance when β < 0. The experimental results in Figures 5b and 6b also show that the scaling factor β may have different values for different features and image databases.

The comparison between the proposed methods (RLTP and SCCLTP with optimal threshold α and β) and the methods in [22] (τ = 5 and β = 0) is given in Table 2. The improved methods, ${\text{RLTP}}_{P,R}^{\text{riu}2}$, and ${\text{SCCLTP}}_{P,R}^{\text{riu}2}$, get an average accuracy improvement of 1% and 0.5% over their original versions, respectively.

Further, we compare the feature extraction complexity of the proposed operators, SCC_OC_RLTP, OC_RLTP and CCLTP, in [22]. The experimental results on TC10 are given in Table 3, where the three different multi-resolutions, *P* = 8 and *R* = 1, *P* = 16 and *R* = 2 and *P* = 24 and *R* = 3 are concentrated together for feature description, as in [22]. The time complexity of computing the two thresholds α and β was not considered in this experiment, because they can be achieved off-line. It is clear that the proposed methods have lower computational complexity than CCLTP.

### 4.2. Experiments for ATR

**Figure 7.**
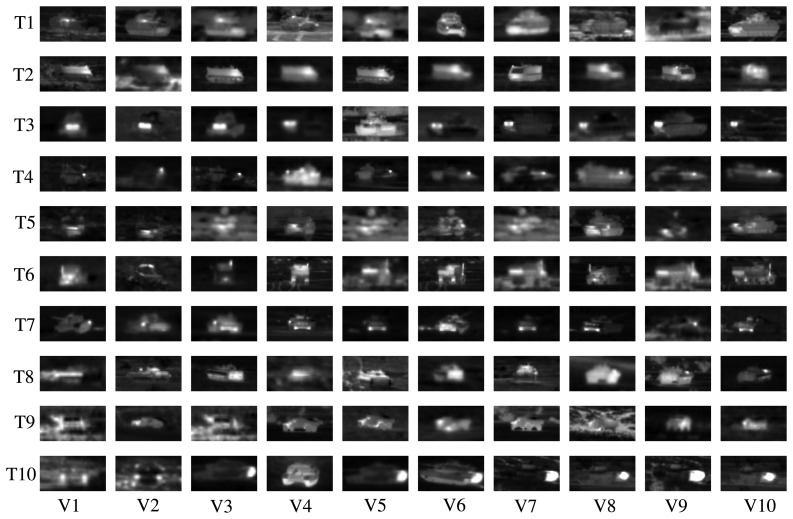
Some infrared chips of the 10 targets (row-wise) in 10 views (column-wise) in the forward-looking infrared (FLIR) dataset.

The same FLIR dataset as [22] is used in this paper for ATR. There are 10 different military targets denoted as *T*1, *T*2, …, T10. For each target, there are 72 orientations, corresponding to aspect angles of 0°, 5°, …, 355°. The dataset contains 437 to 759 images (40 × 75) for each target type, in total 6930 infrared chips. In Figure 7, we show some infrared chips for 10 targets under 10 different views. In the following experiments, the three different multi-resolutions, *P* = 8 and *R* = 1, *P* = 16 and *R* = 2 and *P* = 24 and *R* = 3, are also concentrated together for feature description as in [22].

#### 4.2.1. Comparison of CC_OC_LTP, OC_LTP, OC_LBP and CCLTP

**Figure 8.**
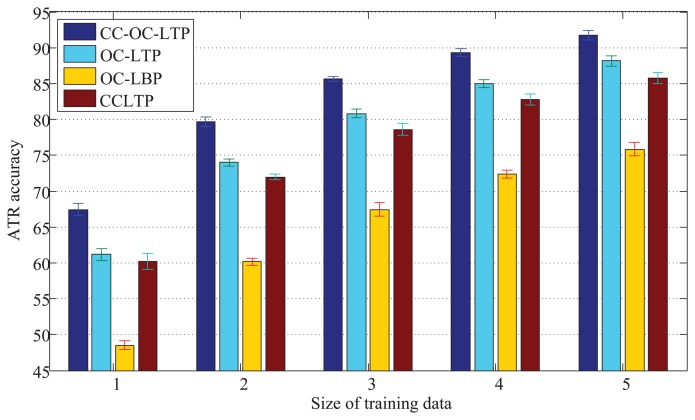
Recognition accuracy comparison for CC_OC_LTP, OC_LTP, OC_LBP and CCLTP.

We evaluate the performance of the operators CC_OC_LTP, OC_LTP, OC_LBP [36] and CCLTP [22] in this section. We randomly chose about 10% (718 chips), 20% (1436 chips), 30% (2154 chips), 40% (2872 chips) and 50% (3590 chips) target chips in each target class as training data. The remaining 90%, 80%, 70%, 60% and 50% images in the dataset are set as testing data, respectively. The mean and variance of the recognition accuracy averaged by 10 trails are given in Figure 8, where CC_OC_LTP, OC_LTP, OC_LBP and CCLTP denote $\text{CO}\_\text{OC}\_{\text{LTP}}_{8,1+16,2+24,3}^{\text{riu}2}$, $\text{CO}\_{\text{LTP}}_{8,1+16,2+24,3}^{\text{riu}2}$, $\text{CO}\_{\text{LTP}}_{8,1+16,2+24,3}^{\text{riu}2}$ and ${\text{CCLTP}}_{8,1+16,2+24,3}^{\text{riu}2}$, respectively. In can be seen from the experimental results that:

- The operators CC_OC_LTP and OC_LTP get better results than CCLTP in [22], and CC_OC_LTP is the best in the four operators.
- With CCP enhancement, the average accuracy improvement of CC_OC_LTP is 4.94% compared with OC_LTP. It further was proven that the CCP method introduced in [22] is effective at improving the performance of the LBP-based methods.
- The OC_LTP gets better recognition performance than OC_LBP [36] and CCLTP [22].
- The CCLTP [22] is better than OC_LBP [36].
- The experimental results also show that CC_OC_LTP, OC_LTP and OC_LBP are robust for infrared ATR, because they are fairly stable in 10 random trials, as CCLTP.

#### 4.2.2. Comparison of RLTP, SCCLTP with LTP and CCLTP, Respectively

**Figure 9.**
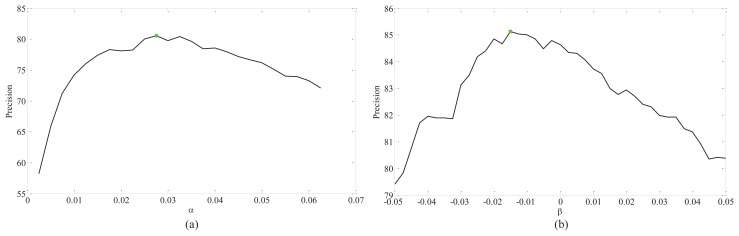
The curve of precision *vs.* α and β for ATR.

In this experiment, we mainly tested the impact of α and β on the RLTP and SCCP for infrared ATR, and the training data and test data are set the same as the above experiment. The curves of the precision *vs.* α and β for ${\text{RLTP}}_{8,1+16,2+24,3}^{\text{riu}2}$ and ${\text{SCCLTP}}_{8,1+16,2+24,3}^{\text{riu}2}$ are given in Figure 9, where the colored boxes in the curves denote that the methods obtain the best performance at that point.

The comparison between ${\text{RLTP}}_{8,1+16,2+24,3}^{\text{riu}2}$ (with optimal threshold α) and ${\text{LTP}}_{8,1+16,2+24,3}^{\text{riu}2}$ (τ = 8) and ${\text{SCCLTP}}_{8,1+16,2+24,3}^{\text{riu}2}$ (with optimal threshold β) and ${\text{CCLTP}}_{8,1+16,2+24,3}^{\text{riu}2}$ in [22] (τ = 8 and β = 0) are given in Tables 4 and 5, respectively. It can be seen that the ${\text{RLTP}}_{8,1+16,2+24,3}^{\text{riu}2}$ gets an average of nearly 3.4% higher performance than ${\text{LTP}}_{8,1+16,2+24,3}^{\text{riu}2}$, and the ${\text{SCCLTP}}_{8,1+16,2+24,3}^{\text{riu}2}$ gets an average of nearly 0.5% higher performance than ${\text{CCLTP}}_{8,1+16,2+24,3}^{\text{riu}2}$. The experimental results show that the introduced schemes are effective for LTP and CCP in infrared ATR.

#### 4.2.3. Comparison of Blocking Methods

In this section, the sCCLTP proposed in [22] was chosen as the testing operator to compare the performance of the two blocking methods given in Figure 4a,b. The training data and test data are set the same as the above experiment. The recognition accuracy averaged by 10 trials is given in Table 6. The experimental results shows that the blocking method introduced in the paper (Figure 4b) gets an average accuracy improvement of 1.3% compared to that of Figure 4a used in [22].

#### 4.2.4. Comparison of Feature Selection

In this experiment, we randomly selected 10% (718 chips), 20% (1436 chips), 30% (2154 chips), 40% (2872 chips), 50% (3590 chips), 60% (4308 chips), 70% (4958 chips) and 80% (5607 chips) target chips in each target class as training data. The remaining 90%, 80%, 70%, 60%, 50%, 40%, 30% and 20% images in the dataset are set as testing data, respectively. The operators $\text{SSC}\_\text{OC}\_{\text{RLTP}}_{8,1+16,2+24,3}^{\text{riu}2}$ and $\text{OC}\_{\text{RLTP}}_{8,1+16,2+24,3}^{\text{riu}2}$ are selected for feature description. Furthermore, the blocking methods in Figure 4b are chosen in the paper. After that, the features of each block and that of the whole image are concentrated together for image description, which are denoted as sSCC_OC_RLTP and sOC_RLTP, respectively. At the same time, the FSDE introduced in [47] is used for feature selection. The selected dimensionalities for sSCC_OC_RLTP and sOC_RLTP are 288, 576, 864, 1152 and 1440 bins, respectively.

The recognition accuracy averaged by 10 trials was given in Tables 7 and 8. It can be seen from the experimental results that:

- The dimensionalities of the selected features are only 6.25%, 12.5%, 18.75%, 25% and 31.25% of sSCC_OC_RLTP (4608) and 12.5%, 25%, 37.5%, 50% and 62.5% of sOC_RLTP (2304).
- It can be seen from Tables 7 and 8 that, with SCCP enhancement, sSCC_OC_RLTP gets higher accuracy than sOC_RLTP.
- The experimental results in Table 7 show that the sSCC_OC_RLTP-1440 (sSCC_OC_RLTP with 1440 dimensionalities by feature selection) gets the best performance when we chose 10%, 20%, 30%, 40% or 50% target chips in each target class as training data and the sSCC_OC_RLTP-1152 (sSCC_OC_RLTP with 1152 dimensionalities by feature selection) gets the best performance when we chose 60%, 70% or 80% target chips in each target class as the training data. For the leave-one-out experiment, sSCC_OC_RLTP-1152 also gets the best results.
- The experimental results in Table 8 show that the sOC_RLTP-576 (sOC_RLTP with 576 dimensionality by feature selection) gets the best performance in the five different cases.
- The results in Tables 7 and 8 also prove that not all of the features in sSCC_OC_RLTP and sOC_RLTP have essential contributions to the operators. The feature selection method FSDE presented in [47] is effective, and it can drop the redundant features effectively.

#### 4.2.5. Comparison of sSCC_OC_RLTP, sOC_RLTP, sCCLTP and SRC-Based Methods

In this section, we compare the performance of the proposed methods, sOC_RLTP and sSCC_OC_RLTP, with sCCTLP introduced in [22] and two SRC-based methods (Sparselab-lasso and SPG-lasso) [16], which are also tested in [22]. The training data and test data are set the same as the above experiment. The dimensionality of 576 for sSCC_OC_RLTP and sOC_RLTP is chosen in the experiment. The recognition accuracies of sSCC_OC_RLTP-576, sOC_RLTP-576, sCCLTP and the sparse-based methods that are averaged by 10 trials are given in Table 9, where we also include the leave-one-out experimental result for each method. It can be seen from the experimental results that:

- The operator sCCLTP gets better performance than the SRC-based methods (SPG-lasso and Sparselab-lasso), which have been verified in [22].
- The performance of sSCC_OC_RLTP-576 is better than sCCLTP and sOC_RLTP-576, while, its dimensionality is far less than that of sCCLTP.
- Because of the lower dimensionality, the time consumed for training and recognition for sSCC_OC_RLTP-576 and sOC_RLTP-576 is also lower than that of the sCCLTP.

**Figure 10.**
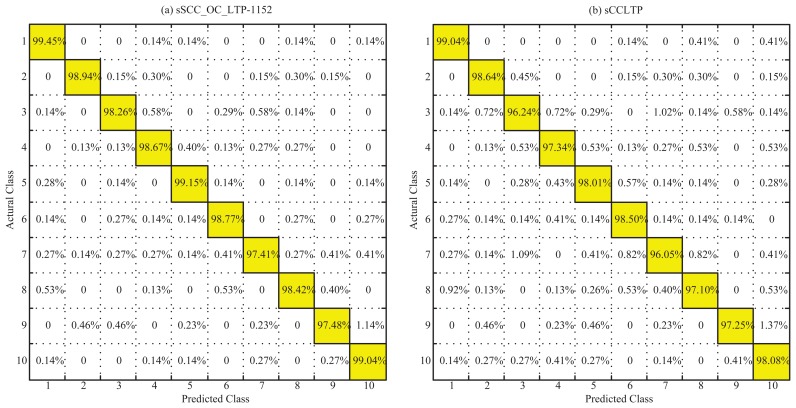
Confusion matrices of the sSCC_OC_RLTP and sCCLTP.

Furthermore, we gave the confusion matrices of sSCC_OC_RLTP-1152 and sCCLTP corresponding to the leave-one-out experiment in Figure 10. The results show that the sSCC_OC_RLTP-1152 result has only one non-diagonal entry greater than 1% (Figure 10a), while sCCLTP has three non-diagonal entries greater than 1% (Figure 10b). On the other hand, all of the diagonal entries of sSCC_OC_RLTP are greater than that of sCCLTP, which shows the better robustness of sSCC_OC_RLTP.

Finally, we give a brief comparison of sSCC_OC_RLTP, sOC_RLTP and sCCLTP [22] on computing complexity. Their complexity mainly contains two aspects: one is the feature extraction complexity, and the other is the training and recognition complexity The experimental results in Table 3 denote that the feature extraction complexity of the proposed methods is lower than that of sCCLTP The training and recognition complexity for the three methods is associated with their dimensionalities according to the dissimilarity measure (chi-square distance). By feature selection, the dimensionalities of the proposed methods may be lower than that of the sCCLTP (1080). The comparison among them is given in Tables 7, 8 and 9. The results proved that the proposed methods can achieve better performance with far less dimensionality than that of the sCCLTP. The feature selection step and the step of obtaining the two thresholds α and β can be implemented off-line. Hence, they do not increase the computing complexity of the real-time recognition of the infrared target.

#### 4.2.6. The Impact of the Gray Variance on the Recognition Performance

In general, the gray values of the target are larger than that of the background for the infrared chips that we chose in the experiments. It is obvious that the gray variance of each chip reflects the contrast between the target and the background. On the one hand, the larger variance denotes greater contrast between the target and its background. On the other hand, larger variance means the target in the chips is easier to recognize. Therefore, such contrast reflects the signal-to-noise ratio of the chips to some extent. In this case, the recognition rates in different variance ranges are able to prove the performance of the different operators. We will further evaluate the methods' performance by the gray variance of the chips.

Firstly, the variance range and the number of chips of each target class is given in Table 10, where min_variance and max_variance denote the minimum and maximum variance of each class. It is clear that the variance range is maximum for the first target class and minimum for the seventh target class. The maximum and minimum of the gray variance are 9.5 and 143.6 for the whole database.

**Figure 11.**
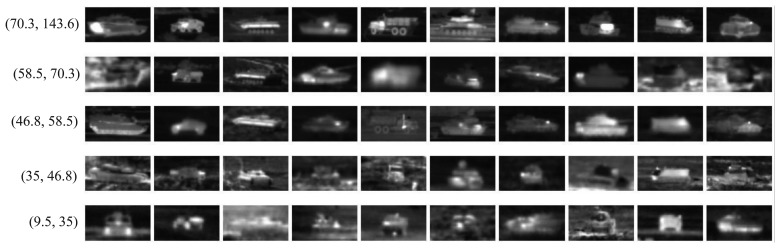
Examples of targets in each variance range.

By gray variance, the chips of each class are classified into five different ranges in this experiment, which are (9.5, 35.0), (35.0, 46.8), (46.8, 58.5), (58.5, 70.3) and (70.3, 143.6). The numbers of chips in each range are given in Table 11. Further, we give an example chip for each target class in different variance ranges in Figure 11. For each range, we randomly selected almost 50% chips in each class as the training data and the remaining as testing data, respectively. The three operators, sSCC_OC_RLTP-576, sOC_RLTP-576 and sCCLTP, are selected for feature description. The recognition rate in each range averaged by 10 random trials is given in Table 12.

It can be seen from Table 12 that the recognition rate is improved gradually with the increase of the gray variance. The same conclusion can also be obtained from the confusion matrices of sSCC_OC_RLTP-1152 and sCCLTP in Figure 10. Whether for sSCC_OC_RLTP-1152 or sCCLTP, the recognition rate of the seventh class is minimal, and that of the first class is maximal. We think the variance range is the main reason.

## 5. Conclusions

This paper presents improved local ternary patterns (LTP) for ATR in infrared imagery. Firstly, the RLTP and SCCP approaches are proposed to overcome the shortcomings of the LTP and CCP, respectively. Combined with the advantage of OC_LBP, SCC_OC_RLTP is further introduced based on RLTP and SCCP. Then, a simple, yet effective, blocking scheme and a feature selection method are introduced to enhance its efficiency for ATR in infrared imagery. Experiments show that the proposed operators can achieve competitive results compared with the state-of-the-art methods.

## Figures and Tables

**Table 1.** Dimensionality comparison.

	(***P*,*R***) = (**8,1**)	(***P*,*R***) = (**16,2**)	(***P*,*R***) = (**24,3**)
OC_LBP*_P,R_*	32	64	96
OC_RLTP*_P,R_*	64	128	192
SCC_OC_RLTP*_P,R_*	128	256	384

**Table 2.** Classification accuracy (%) on the TC10 and TC12 texture sets.

	**(*P*, *R*)** = **(8, 1)**				**(*P*,*R*)** = **(16,2)**				**(*P*, *R*)** = **(24, 3)**			

	**TC10**	**TC12**		**Average**	**TC10**	**TC12**		**Average**	**TC10**	**TC12**		**Average**
		
		***t***	***h***			***t***	***h***			***t***	***h***	
${\text{LTP}}_{P,R}^{\text{riu}2}$	94.14	75.87	73.95	81.32	96.95	90.16	86.94	91.35	98.20	93.58	89.42	93.73
${\text{RLTP}}_{P,R}^{\text{riu}2}$	93.78	77.71	75.58	82.36	97.42	91.02	88.70	92.38	98.91	94.91	92.59	95.47
${\text{CCLTP}}_{P,R}^{\text{riu}2}$	96.87	86.96	88.10	90.64	98.20	94.53	94.46	95.73	98.75	95.67	92.91	95.77
${\text{SCCLTP}}_{P,R}^{\text{riu}2}$	97.37	87.43	88.52	91.11	98.52	95.14	94.75	96.14	98.83	96.11	93.77	96.24

**Table 3.** The average feature extraction time per image on TC10.

	**SCC_OC_RLTP**	**OC_RLTP**	**CCLTP**
Average feature extraction time (s)	0.012	0.009	0.013

**Table 4.** Accuracy of infrared ATR (%) for RLTP and LTP under different training datasets.

**Methods**	**10%**	**20%**	**30%**	**40%**	**50%**
${\text{LTP}}_{8,1+16,2+24,3}^{\text{riu}2}$	51.58	62.67	69.12	73.53	76.71
${\text{RLTP}}_{8,1+16,2+24,3}^{\text{riu}2}$	54.22	65.90	72.81	77.14	80.48

**Table 5.** Accuracy of infrared ATR (%) for SCCLTP and CCLTP under different training datasets.

**Methods**	**10%**	**20%**	**30%**	**40%**	**50%**
${\text{CCLTP}}_{8,1+16,2+24,3}^{\text{riu}2}$	60.23	71.97	78.61	82.78	85.74
${\text{sCCLTP}}_{8,1+16,2+24,3}^{\text{riu}2}$	60.84	72.48	79.16	83.28	86.13

**Table 6.** Accuracy of infrared ATR (%) for the two blocking methods under different training datasets.

**Blocking Methods**	**10%**	**20%**	**30%**	**40%**	**50%**
Figure 4a	66.50	79.05	85.88	89.80	92.25
Figure 4b	68.61	80.32	86.79	91.33	92.81

**Table 7.** Accuracy of infrared ATR (%) for sSCC_OC_RLTP by FSDE [47] under different training datasets.

**Dimensionality**	**10%**	**20%**	**30%**	**40%**	**50%**	**60%**	**70%**	**80%**	**Leave-One-Out**
288	70.71	82.28	87.79	91.15	93.42	94.48	95.48	96.14	97.94
576	71.64	83.06	88.74	91.79	94.03	95.16	96.08	96.72	98.34
864	72.12	83.50	89.20	92.30	94.43	95.32	96.24	96.85	98.43
1152	72.79	84.50	89.61	92.54	94.56	**95.57**	**96.46**	**96.91**	**98.61**
1440	**72.91**	**84.77**	**89.70**	**92.59**	**94.58**	95.47	96.33	96.88	98.37

**Table 8.** Accuracy of infrared ATR (%) for sOC_RLTP by FSDE [47] under different training datasets.

**Dimensionality**	**10%**	**20%**	**30%**	**40%**	**50%**	**60%**	**70%**	**80%**	**Leave-One-Out**
288	67.83	79.98	86.19	89.58	92.27	93.48	94.71	95.23	97.50
576	**69.24**	**81.61**	**87.57**	**90.97**	**93.29**	**94.32**	**95.60**	**96.36**	**98.11**
864	68.22	80.61	86.99	90.41	92.91	94.05	95.17	95.96	97.81
1152	68.18	80.56	86.79	90.27	92.76	93.97	95.05	95.74	97.66
1440	68.40	80.75	87.04	90.37	92.87	94.10	95.22	95.90	97.78

**Table 9.** Accuracy of infrared ATR (%) for the six methods under different training datasets.

**Methods**	**10%**	**20%**	**30%**	**40%**	**50%**	**60%**	**70%**	**80%**	**Leave-One-Out**
sSCC_OC_RLTP-576	71.64	83.06	**88.74**	**91.79**	**94.03**	**95.16**	**96.08**	**96.72**	**98.34**
sOC_RLTP-576	69.24	81.61	87.57	90.97	93.29	94.32	95.60	96.36	98.11
sCCLTP	66.50	79.05	85.88	89.80	92.25	93.55	94.64	95.29	97.63
SPG-lasso	75.45	**84.43**	88.51	91.10	92.76	93.87	94.54	95.23	96.87
Sparselab-lasso	**75.65**	83.95	87.95	90.24	91.86	93.04	93.82	94.43	95.84

**Table 10.** The variance range of each target class.

**Target Class**	**1**	**2**	**3**	**4**	**5**	**6**	**7**	**8**	**9**	**10**
number of chips	729	660	691	753	702	733	735	759	437	731
min_variance	17.7	18.9	19.9	12.1	13.0	9.5	12.7	13.1	23.2	13.1
max_variance	143.6	102.3	107.1	115.1	121.7	92.9	82.0	118.8	119.9	104.4

**Table 11.** Number of chips in each range.

**Variance Range**	**(9.5, 35.0)**	**(35.0, 46.8)**	**(46.8, 58.5)**	**(58.5,70.3)**	**(70.3,143.6)**
number of chips	1409	1747	1478	1009	1287

**Table 12.** The recognition rate in each range averaged by 10 random trials.

**Variance Range**	**(9.5, 35.0)**	**(35.0, 46.8)**	**(46.8,58.5)**	**(58.5,70.3)**	**(70.3,143.6)**
sSCC_OC_RLTP-576	92.54	93.23	93.85	94.61	95.88
sOC_RLTP-576	91.88	92.39	93.02	93.45	94.17
sCCLTP	90.03	91.39	92.08	92.94	93.62
